# Effects of small extracellular vesicles derived from normoxia- and hypoxia-treated prostate cancer cells on the submandibular salivary gland epithelium *in vitro*

**DOI:** 10.1080/21688370.2024.2347062

**Published:** 2024-05-09

**Authors:** Ana Špilak, Andreas Brachner, Heinz-Peter Friedl, Adrián Klepe, Christa Nöhammer, Winfried Neuhaus

**Affiliations:** a Competence Unit Molecular Diagnostics, Center for Health and Bioresources, AIT - Austrian Institute of Technology GmbH, Vienna, Austria; b Faculty of Medicine and Dentistry, Danube Private University, Krems, Austria

**Keywords:** A253, biological barriers, blood-saliva barrier, DU145, EV, HTB-41 B2, hypoxia, normoxia, PCa, prostate cancer, sEV, small extracellular vesicles, uptake

## Abstract

Small extracellular vesicles (sEVs) are an important part of intercellular communication. They are phospholipid bilayer particles that carry active biomolecules such as proteins, various nucleic acids, and lipids. In recipient cells, sEVs can alter cellular functions, including cancer development and premetastatic niche formation in distant organs. Moreover, sEVs can carry cancer-specific features, which makes them promising biomarker candidates. However, the interactions of sEVs with biological barriers and consequences thereof, are not clarified yet. The blood-saliva barrier is crucial for preventing the entry of pathogens and (in)organic substances into the bloodstream, as well as molecule filtration from blood to saliva. The effects of brain derived DU145 prostate cancer (PCa) sEVs on a human submandibular salivary gland barrier (SSGB) *in vitro* were investigated. Small EVs were harvested from normoxic (N, atmospheric O_2_) or hypoxic (H, 1% O_2_) conditions, fluorescently labeled with CellTracker^TM^ Orange and thoroughly characterized. HTB-41 B2 cells were used as SSGB model cultured on 24-well ThinCert® inserts. After model optimization indicating effects of serum and serum-sEVs on barrier properties, PCa sEVs were applied to the basolateral (blood) side in either 10% serum, or serum-free conditions, and barrier integrity was continuously monitored for 40 hours. This study found that H and N PCa sEVs were uptaken by the SSGB *in vitro* model in similar quantities regardless of the media composition in the basolateral compartment. Permeation of fluorescent PCa sEVs into the apical compartment was not detectable with the applied methods. However, treatment with H and N sEVs under different serum conditions revealed distinct molecular clusters after hierarchical analysis of mRNA data measured by high-throughput qPCR, which were partly reflected at the protein level. For example, serum-reduction dependent decrease of barrier properties was accompanied with the decrease of CDH1 or Claudin-7 expression. Interestingly, the presence of H sEVs significantly increased the number of sEV-sized particles in the apical compartment of the SSGB model compared to basolaterally added N sEVs. This functional effect on the number of particles in the saliva (apical) compartment induced by different sEVs applied in the blood (basolateral) compartment might be a new approach to understand one possible mechanism how differences of salivary EVs might occur which then could be used as biomarker.

## 1. Introduction

The epithelial cell layers of the oral mucosa and salivary glands are forming the so-called blood-saliva barrier (BSB).^1^ This barrier is crucial in preventing the viral, bacterial and (in)organic substance entry into the bloodstream. The BSB function also includes electrolyte exchange and molecule filtration from blood to saliva, a process which is being utilized for potential liquid biopsy applications, such as, for example, determining cortisol levels in saliva instead of blood.^2^ New potential biomarkers in saliva could be found in extracellular vesicles (EVs), since their composition varies based on individual (patho)physiological state.^3,4^

EVs are a miscellaneous group of nano-sized phospholipid bilayer spheres with a hydrophilic inner core. These heterogeneous particles are released by all cells and carry diverse repertoire of molecular cargo such as nucleic acids, proteins and lipids. EV cargo is dependent of their cell of origin and may include signaling molecules and enzymes. Consequently, EVs are found in virtually all body fluids such as blood, saliva, cerebrospinal fluid and urine, where they have a unique composition, largely dependent on the cells of origin.^5^ EVs have been shown to be important mediators of cell communication in very diverse array of physiological processes, from nervous system development to exercise.^6,7^ Additionally, they have been shown to play a part in pathological processes during, for example, cancer, cardiovascular and neurodegenerative disease.^8–12^

Due to their ability to reflect the current metabolic state of the parental cell EVs are becoming a valuable tool for diagnostics.^13–15^ Based on their size and synthesis route, there are three main types of EVs. Apoptotic bodies are the largest vesicles ranging up to several micrometers and are the remnants of apoptotic cells, containing fragmented DNA and organelles.^16,17^ Microvesicles are typically 100–1000 nm in size and are formed by outward budding of the plasma membrane.^18^ The smallest vesicles are called small EVs (sEVs) or exosomes, typically ranging between 50–200 nm in size and are formed by inward budding of the endosomal membrane and formation of multivesicular bodies. When multivesicular bodies fuse with the plasma membrane, these sEVs, rich in proteins, lipids and various nucleic acids are released into the extracellular space.^19,20^

Small EVs can be uptaken by other cells and alter cell function and behavior of the receiving cell. Under pathological conditions, sEVs have been shown to be involved in premetastatic niche (PMN) formation.^9,21,22^ Besides their role in cancer metastasis, it was already shown that salivary EVs carry molecular signals from blood^23,24^ and even brain following traumatic brain injury.^8^ In addition, it was discovered in mouse models that EV-like particles carrying signatures from lung cancer cells can be found in saliva,^23^ and that salivary sEVs from tumor-bearing mice have the potential to transmit suppressive signals to immune cells, which could have a role in cancer development.^25^ These findings confirm that signatures from cancer cells on EVs are transported via circulation to distal sites in the body, but the processes and interactions behind EV transport from circulation into saliva and EV secretion from salivary gland cells have not been identified yet.

Prostate cancer (PCa) ranks as the second most common cancer in men.^26^ The conventional diagnostic approach is based on determination of the highly variable prostate specific antigen (PSA).^27^ Nevertheless, novel biomarkers and therapy targets are investigated for diagnosis and treatment, including potential biomarkers present in sEVs.^13,28,29^

The hypoxic microenvironment in solid tumors is a known driver of cancer progression, having a role in increasing tumor survival, invasion, and metastasis.^30^ Hypoxia was shown to upregulate the release of sEVs in several cancer and non-cancerous cell lines, therefore lack of oxygen could be a universal trigger of sEV release.^31–34^ One possible mechanism for enhanced sEV secretion is activation of the hypoxia inducible factor (HIF) pathway,^33,35^ that is also activated in hypoxic PCa.^30^

The aim of this study was to investigate the effects of PCa derived sEVs on a human submandibular salivary gland epithelium barrier (SSGB) model *in vitro*, including a comparison between sEVs from hypoxic and normoxic conditions. Standardized BSB models mimicking *in vivo* BSB characteristics are still limited.^1,36^ Nevertheless, recently an improved submandibular salivary gland epithelium barrier (SSGB) model based on a single cell clone of the tumor-derived cell line HTB-41^TM^ (A253) was described. In this SSGB model paracellular tightness properties were improved and were more similar to *in vivo* conditions.^37^ Thus, this model was chosen for the presented studies. The interactions between fetal bovine serum (FBS) EVs present in the complete growth media and the SSGB (or the transport of FBS EVs across the SSGB) are not known. To ensure the validity of our model and to investigate conditions being presumably physiologically more relevant, we first conducted comparisons between serum-free conditions in both the apical and basolateral compartments, as well as conditions with apical serum-free media and basolateral FBS-containing media. We show that the complete absence of serum compromised barrier integrity the most, but basolateral FBS helped in maintaining barrier integrity. Therefore, it was concluded that serum-free apical and FBS containing basolateral media are the best media conditions for planned EV studies. Then, small EVs were applied to the basolateral compartment of ThinCert® barrier models, corresponding to the blood side in the physiological situation, hence simulating the interaction of sEVs found in blood with SSGB cells. With sEVs derived under normoxic and hypoxic conditions from DU145 PCa cells, we mimicked different cancer metastatic potentials. Uptake of sEV, changes in the barrier cells at the cellular and molecular level, and the release of sEVs on the apical (salivary) side of the model were determined. In summary, this study describes the effect of different PCa sEV populations on the SSGB barrier.

## 2. Material & methods

### 2.1. HTB-41 B2 salivary gland barrier model

HTB-41 clone B2 was isolated and established from HTB-41 (A253), a tumor-derived submandibular salivary gland cell line.^37^ The cell line was maintained in McCoy’s 5A media (Thermo Fisher, Waltham, MA, USA; 16600–082) supplemented with 1% Pen/Strep (Penicillin/Streptomycin, Merck, Darmstadt, Germany; A2213) and 10% FBS (Fetal Bovine Serum, Sigma-Aldrich, St. Louis, MO, USA; F9665). Cells were split once per week and seeded at a density of 8 × 10^3^ cells/cm^2^. Media changes were performed every 2–3 days. For salivary gland barrier studies, cells were seeded at a density of 8 × 10^4^ cells/cm^2^ in the apical compartment of 24-well ThinCert® inserts (Greiner Bio-One GmbH, Kremsmünster, Austria; 662641). At the apical side 300 µL and basolaterally 900 µL of media were used. Media were exchanged every 2–3 days, and after 14–15 days cells reached maximum barrier tightness as established by Lin et al., 2020^9^ and were used for experiments. Initial TER values assessed with the CellZScope device as described in 2.2 were 463,7 ± 35,5 Ω*cm^2^, in case of uptake experiments TER values were measured with chopsticks and corresponded to 867,8 ± 172,4 Ω*cm^2^. Cells were cultivated at 37°C, in a 95% humidified atmosphere containing 5% CO_2_.

### 2.2. Barrier integrity assessment

In order to assess barrier integrity of HTB-41 B2 SSGB models, transepithelial electrical resistance (TER) [Ohm/cm^2^] and cell layer capacitance (CAP) [µF/cm^2^] was assessed with a CellZscope device (NanoAnalytics). Exception were uptake experiments, where TER was assessed with a manual chopstick electrode (WPI, #STX2) coupled to an EVOM3 Epithelial Volt Ohm Meter (WPI). Prior to the first TER measurement, media were exchanged as described in 2.9 and equilibrated for 40 minutes at room temperature. The chopstick electrode was first disinfected in 70% EtOH for two to five minutes and then equilibrated in the fresh media for at least 30 minutes. TER values are displayed as Ω*cm^2^, calculated by subtraction of the mean value of blank inserts and multiplication with the growth surface area of the used inserts (0.336 cm^2^). For monitoring the barrier properties over a longer period, the CellZScope device was used. In brief, ThinCert® inserts were added to the device in their maintenance media (McCoy++) and incubated for 2-3 h to equilibrate the system temperature in the incubator. Then media were exchanged for the respective experimental media and the cell layers’ impedance was measured hourly in the incubator for the following 40 hours. The second measurement (1 h after start of incubation) was used for data normalization, in order to ensure complete equilibration of the system. Experimental media were always serum-free in the apical compartment (McCoy, 1% Pen/Strep) unless stated otherwise. Basolateral media were either serum-free, with 10% FBS or 10% sEV-depleted FBS in condition optimization experiments and serum-free or 10% FBS when sEVs were added in physiological concentrations.^38^ Small EV depleted FBS was prepared by ultrafiltration, using Amicon-15 ultrafiltering tubes (UFC810096, Sigma-Aldrich). For the final TER [Ω x cm^2^] and CAP values [µF/cm^2^] blank inserts (without cells) were subtracted for each time point, and values were calculated according to the algorithm of the CellZscope Software (Version 2.2.4, nanoAnalytics GmbH).

### 2.3. DU145 prostate cancer cells for sEV isolation

DU145, human prostate adenocarcinoma cells were maintained in DMEM (Sigma, D5796) supplemented with 10% FBS, 1% Sodium pyruvate solution (S8636, Merck Life) and 1% Pen/strep. Splitting was conducted with Trypsin/EDTA (PAN Biotech; P10-023100) in 1:6 or 1:8 ratios two times per week and cells were maintained in 15 mL media in T75 cell culture flasks (658175, Greiner BioOne). For the sEV isolation, cells were seeded onto larger cell culture dishes (PD145; 639160, Greiner Bio-One). When cells reached 75–80% confluency they were washed twice with 5 ml of serum-free maintenance medium. After washing, cells were incubated in 25 ml of serum-free maintenance medium for 48 h at 37°C, in a humidified atmosphere containing 5% CO_2_. In order to collect sEVs under hypoxic conditions, cells were incubated accordingly in a hypoxia chamber (Biospherix, BIOS-C474 with ProOx C21 O_2_/CO_2_ Controller) in a humidified atmosphere with 1% O_2_ and 5% CO_2_.

### 2.4. Small EV isolation

**Figure 1.**
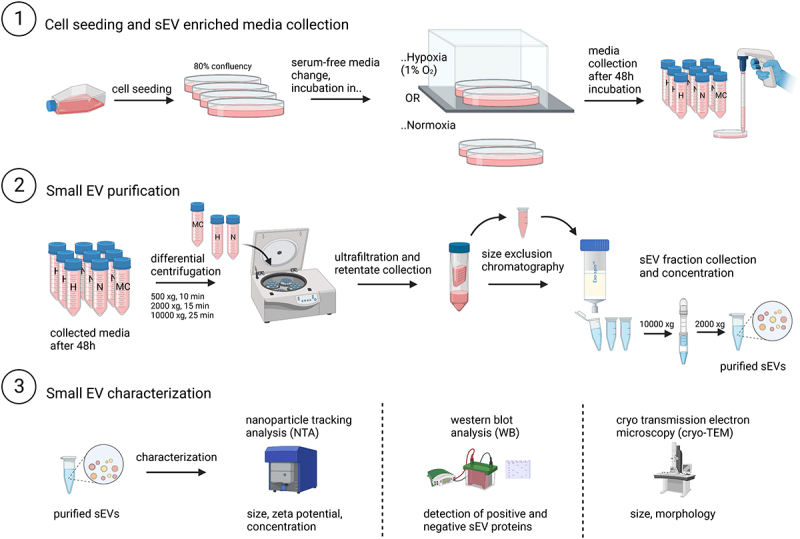
Small EV collection, isolation, and characterization scheme. H, hypoxia; N, normoxia; MC, media control.

After incubation of DU145 cells with serum-free medium, cell culture supernatants were collected and subjected to a series of centrifugation steps: 300× g for 10 minutes, 2000× g for 15 minutes and 10,000× g for 25 minutes with 50 ml centrifugation tubes being exchanged after every centrifugation step. In the meantime, cells were lysed for western blot analysis as described in 2.6 and detached with Trypsin/EDTA for viability check with trypan blue. High viability of cells used for sEV collection is crucial to avoid contamination with other types of EVs such as apoptotic bodies. Media centrifugation steps were followed by ultrafiltration and size exclusion chromatography (SEC) performed according to the manufacturer’s instructions. Shortly, after the medium was centrifuged and depleted of cells, apoptotic bodies and larger microvesicles, the cleared supernatant was subjected to ultrafiltration in Amicon-15 ultrafiltering tubes (UFC810096, Sigma-Aldrich) in order to concentrate the contained EVs. Retentate volume was collected and applied to SEC columns (EX04, CellGS). Small EV rich fractions (7–13) were collected in DPBS in protein low binding tubes (Eppendorf 022,431,102). These fractions were then concentrated with Amicon-Ultra 0.5 centrifugal filter tubes (UFC510096, Sigma-Aldrich) at 10,000 ×g until a final volume of 100–200 µL was reached. As negative control in uptake experiments, serum-free media were incubated in petri dishes in parallel with the cell culture dishes and under both oxygen conditions, and potentially present sEV-like particles were isolated using the identical procedure as used for sEV isolation. Small EV collection, isolation and characterization scheme is summarized in Figure 1.

### 2.5. Nanoparticle tracking analysis (NTA)

NTA (ZetaView® Quatt, PMX420, Particle Metrix GmbH) was used to determine sEV concentration, zeta potential and size distribution. Isolated and concentrated sEV samples were diluted in DPBS to achieve 100–200 particles/frame. Apical media samples from three inserts were pooled in order to ensure enough volume for analysis. Samples were collected in 2 mL centrifugation tubes and depleted of possible cell debris and larger vesicles with a series of centrifugation steps (300× g for 5 minutes, 2000× g for 10 minutes and 10,000× g for 15 minutes) before measuring on NTA. For measurement in scatter mode with a 488 nm laser, sensitivity 75 and shutter 100 was set. Particles were measured in 11 positions. Labelled CTO+ sEVs were additionally measured in low bleaching fluorescence mode NTA with 520 nm laser with 550 nm filter cutoff, sensitivity 92, shutter 95 and minimum particle brightness 25.

### 2.6. Western blotting and EV proteomics

Cell lysis, polyacrylamide gel electrophoresis (PAGE) and Western blotting were done with minor modifications as described before.^39^ Briefly, cells were washed two times with ice-cold DPBS and lysed in RIPA buffer supplemented with a protease-(Roche, cOmplete ULTRA tablets mini 05,892,970,001) and phosphatase-(Roche, PhosSTOP 04,906,837,001) inhibitor cocktail. After 20-minutes incubation on ice, cells were scraped, and the collected cell lysates sonicated for 3 seconds with a sonicator (1 pulse/second, 30% amplitude) and stored at −80°C until use. To determine the protein concentration of cell lysates and sEV samples, a Pierce BCA^TM^ Assay Kit (23225, Thermo Fisher Scientific) or Pierce MicroBCA^TM^ Assay Kit (23235, Thermo Fisher Scientific) were used respectively. Both kits were used as per the manufacturer’s instructions. Small EV samples measured with the MicroBCA assay were lysed in 2% SDS in DPBS as an assay buffer. For SDS-PAGE, 10% gels were used, and 10 µg of protein sample mixed and boiled with Laemmli buffer was loaded per lane. Immunoblotting onto polyvinylidene difluoride (PVDF) membranes was done in a BioRad tank blotter at 4°C, overnight. Membranes were blocked with 5% skimmed milk in DPBS supplemented with 1% Tween®20 for 2 h. The same buffer was used for the application of primary antibodies against Alix, CD81, CD9, GM130, ERK1/2, phospoERK1/2 (overnight, 4°C), ß-actin and complementary secondary antibodies (2 h, room temperature). Antibodies are listed in the Supplementary Table S2. After membrane washing, bands were visualized with Clarity Western ECL-solutions (170–5060, Bio-Rad Laboratories) using a ChemiDoc Touch Imaging System (170–8370, Bio-Rad Laboratories). Blots were visualized and analyzed with the ImageJ/Fiji software.

For proteome identification, sEVs stored at −80°C were precipitated in ice-cold acetone at −20°C overnight. Subsequently, sEV precipitates were centrifuged, washed with 80% acetone in MQ, and allowed to air-dry. Precipitated proteins from sEVs were stored at −80°C until sample preparation for liquid-chromatography-mass spectrometry (LC-MS) analysis. Sample processing, LC-MS analysis, raw data preparations with the identified hits were performed by the Mass Spectrometry Facility at the Max Perutz Labs Vienna as described in their previous publication.^40^ After identifying all proteins in the database search based on their UniProt ID, to be more stringent, valid proteins were set to be at least three peptides after filtering out the contaminant proteins. 3093 proteins were primarily found.

### 2.7. Cryo-transmission electron microscopy (Cryo-TEM)

Small EVs were isolated as described in 2.4. For imaging, 4 µl of sEV suspension in DPBS was loaded per copper grid with 2 µm pores. Excess liquid was removed with automated tapping on filter paper and samples were then snap-frozen in liquid ethane with an automated plunge freezer. Frozen grids were fixed into holders and stored in liquid nitrogen until imaging. Images were recorded with a 150.000-fold magnification (pixel size 0.9719 Å) on a cryo-TEM, Glacios™, Thermo Fisher Scientific.

### 2.8. Small EV labeling with CellTracker® Orange (CTO)

For uptake experiments sEVs were labeled with CellTracker® Orange CMRA (CTO, C34551, Invitrogen). A 10 mM CTO in DMSO stock solution was freshly diluted to 20 µM in DPBS, and equal volumes of CTO dilution and sEV suspension were mixed and incubated light protected with gentle shaking for 3 h at 37°C. After labeling, sEVs were washed with DPBS two times as follows: 20-fold volume of the initial dye volume was added to sEV sample in steps of 500 µL and centrifuged in 100 kDa Amicon-Ultra 0.5 filter tubes at 10,000 ×g. The same labeling procedure was done for the media control samples.

### 2.9. DU145 sEV addition to the HTB-41 B2 barrier model

Prior to sEV addition, cells were washed with serum-free medium twice. Apically 300 µL of medium was used for washing, basolaterally the inserts used for serum-free conditions were submerged in serum-free medium. After washing experimental media were added in the new plate or in the cellZscope device and cells were incubated for 40 h. To assess the effect of different media composition and sEVs on TER and CAP a cellZscope device for continuous monitoring was used, as described in 2.2. For sEV treatment a physiological concentration of 1 × 10^10^ sEVs/mL was used.^38^ Corresponding volume of the DPBS or isolated media control (as described in 2.4 and 2.8) served as negative control. Small EVs were always added in the basolateral compartment corresponding to blood side of this barrier model. Apical media in the sEV treatment were serum-free and basolateral media were either serum-free or contained 10% FBS. For uptake studies, TER measurement was done with chopstick electrodes at the start and after the 40 h incubation with CTO labeled sEVs. Additionally, besides sEV and untreated samples, media labeling controls were added in experiments where labeled sEVs were used. After 40 h, apical and basolateral media were collected, and particles were analyzed by NTA. The cells were either lysed for molecular analyses or detached for uptake measurement. For uptake assessment each insert was washed with DPBS twice. After washing each insert was incubated with 100 µL of Trypsin/EDTA for 15 minutes at 37°C. Then the cells were resuspended in additional 200 µL of maintenance medium and fluorescence was measured at 565 nm (PE channel) with a CytoFlex flow cytometer (Beckman Coulter).

### 2.10. RNA isolation and cDNA synthesis

After the treatments, three HTB-41 B2 insert samples of the same condition were lysed and pooled. For lysis, RNA and protein isolation the “All prep DNA/RNA/Protein” mini kit (Qiagen 80,004) was used according to manufacturer´s instruction. RNA concentrations, 260/280 nm and 260/230 nm ratios were determined with a Nanodrop 2000c spectrophotometer (Thermo scientific). cDNA synthesis was done with Reverse Transcriptase kit (4368814, Applied Biosystems). An input of 250 ng total RNA was used in a 20 µL cDNA reaction mix. The cDNA samples were stored at −80°C.

### 2.11. High-throughput qPCR barrier chip

Prior to qPCR chip analysis, all target sequences were pre-amplified in a single-tube reaction using the HotStar qPCR reaction mix (Qiagen), with 1.5 µl cDNA input and 18 amplification cycles. Aliquots of the pre-amplified DNA samples were loaded in qPCR reaction mixes (TaqMan gene expression master mix, ABI) on a 96 × 96 gene expression chip (Fluidigm) and run on a BioMark HD system (Fluidigm), facilitating the detection of 96 target sequences in 96 samples. Samples were loaded as duplicates, a non-template control (H_2_O) was included.

Threshold cycle (Ct) values were normalized to the endogenous housekeeping gene B2M and relative quantification was performed based on the comparative 2^−ΔCt^ method. Differential gene expression was visualized in a heatmap of log2-fold changes normalized to B2M.

### 2.12. Statistics and data analysis

Results are shown as arithmetic means ± standard deviation (SD) or ± standard error of the means (SEM) unless otherwise indicated. Analyses of automated cell monitoring (TER, CAP) were done with the CellZScope software (Version 2.2.4, nanoAnalytics GmbH). Plots and statistical analyses were done with GraphPad Prism 8.0 (GraphPad Software, USA) and Microsoft Excel. Flow cytometry results were processed in Kaluza Analysis software 2.1 (Beckman Coulter), where median fluorescence intensity values were calculated. Heatmaps of high-through qPCR Chip were done in Qlucore Omics explorer 3.8. Density values of proteins detected by western blotting were acquired with ImageJ/Fiji with the “Analyze Gels” function. Statistical differences were calculated using Student’s t-test or one-way ANOVA followed by pairwise Holm-Šidak’s multiple comparison post hoc tests. For statistical comparisons, an α-value of 0.05 was used. Changes were considered statistically significant at a value of *p* < 0.05.

## 3. Results

### 3.1. Optimization of the experimental conditions for sEV studies reveals serum-dependent effects on the tightness of the salivary gland epithelium model

**Figure 2.**
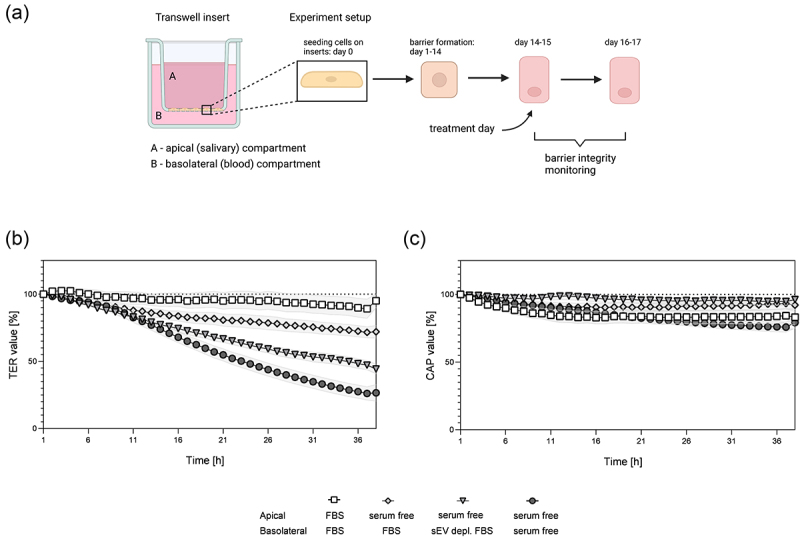
Effect of different serum composition in media in ThinCert® insert model. (a) Schematic representation of an insert with indicated apical and basolateral compartment and experimental workflow to obtain the HTB-41 B2 SSGB model. Media used for treatment on apical (a) and basolateral (b) side of the insert was either serum free, supplemented with 10% fetal bovine serum (FBS), or 10% sEV depleted FBS. Effects of different media composition were recorded with a CellZscope device for continuous monitoring, where (b) transepithelial electrical resistance (TER) and (c) capacitance of cell layers (CAP) were measured in 1 h intervals. Data were normalized to the timepoint 1 h after sEV addition. (b,c) data are represented as mean ± SEM, *N* = 3–5, *n* = 5–14. In table S3 (TER) and table S4 (CAP) statistically significant differences for all conditions are listed.

Normal maintenance medium during HTB-41 B2 barrier development is supplemented with 10% fetal bovine serum (FBS) on both sides of the ThinCert® insert (Figure 2(a)). However, the presence of serum in both compartments of the insert is not physiologically accurate, as saliva does not contain serum components such as albumin in significant concentrations. Small EVs present in FBS, albumin and other serum constituents can also act as contaminants and present a challenge for later analysis due to higher background and possible interactions with tested components. To optimize experimental conditions, serum-free media were applied in the apical (salivary) compartment of the SSGB model. In the basolateral (blood) compartment either 10% FBS, sEV depleted serum, or serum-free media were tested. As control, normal maintenance medium (10% FBS) was used on both sides of the model (Figure 2). Initial TER values at timepoint 0 were 463,7 ± 35,5 Ω*cm^2^, which were in the expected range for this model.^9^ Interestingly, while maintaining cells in basolateral serum-free media were the most detrimental for TER (26,6% of initial value ±6,2), differences were observed also between basolateral FBS (72% of initial value ±4,7) and sEV depleted FBS (44,3% of initial value ±3,6)(Figure 2). The strongest decrease of TER was – as expected – observed between complete 10% FBS medium and serum-free medium on both sides of the insert, but additionally also with the difference only between basolateral compartments − 10% FBS and serum-free (Table S3). CAP of the cell layer remained similar in FBS (92,1% of initial value ±4,6) and sEV depleted FBS (96,2% of initial value ±3,3) conditions and was even higher under these conditions compared to complete FBS control (apical and basolateral FBS, 83,2 of initial value ±6). CAP was mostly affected in serum-free treatment (79.3% of initial value ±4,3). Significant differences were observed between apical serum-free media in all conditions and basolateral EV depleted FBS vs. serum-free as well as between FBS vs. serum-free basolateral condition (Table S4). To summarize, barrier integrity measurements, especially TER values showed that basolateral media containing different serum fractions have significant effects on barrier integrity.

### 3.2. Characterization of sEVs derived from a PCa cell line under hypoxic and normoxic conditions

**Figure 3.**
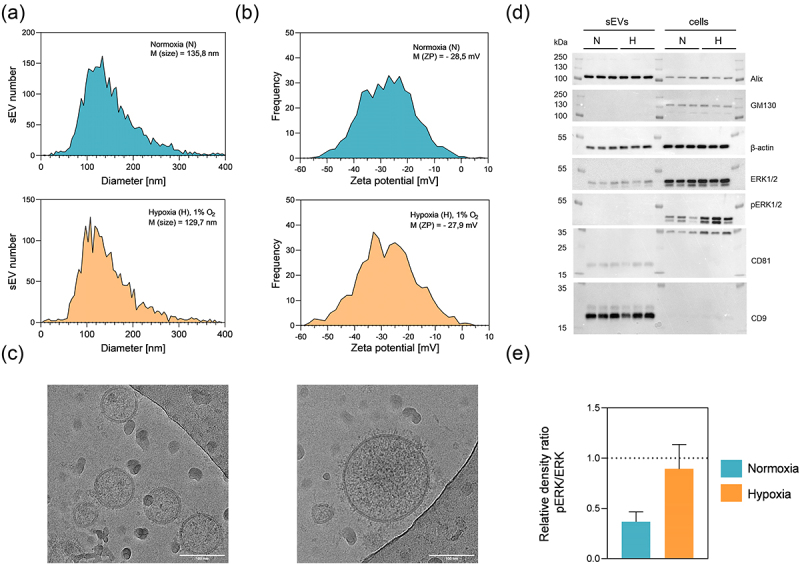
Characterization of sEvs derived from DU145 cells in hypoxic (H) − 1% O_2_ or normoxic (N) – atmospheric O_2_ conditions. Shown are representative graphs of (a) size distribution with indicated median (M (size)) and (b) zeta potential with indicated mean ZP (M (ZP)) of H and N sEvs measured with nanoparticle tracking analysis (NTA). c) cryo-TEM image of DU145 N sEvs. Scale bar = 100 nm. (d) Western blot analysis confirmed the presence of β-actin, sEV enriched (Alix, CD9, CD81) and absence of a sEV negative marker (GM130), as well as hypoxia regulated phosphorylation of ERK1/2. (e) Relative levels of ERK proteins were calculated as described in 2.11. *N* = 3 of pooled samples, *n* = 3 ± SEM.

Small EV donor DU145 cells were subjected to normoxic (N) – atmospheric O_2_ and hypoxic (H) − 1% O_2_ conditions 48 h before sEV collection. Isolation and characterization methods were chosen according to MISEV guidelines for extracellular vesicle studies.^41^ Before application of DU145 sEVs on the barrier model, sEVs were thoroughly characterized. Size and concentration determined with NTA were comparable between N and H sEVs (Figure 3, Table S1). Similarly, zeta potential analysis did not reveal differences between sEV populations, but it confirmed the expected negative zeta potential.^42^ Protein expression profile was determined based on sEV enriched proteins that were selected.^41^ Western blot confirmed the presence and enrichment of Alix, CD9, CD81 in sEVs, while negative marker GM130 was only present in cell lysates (Figure 3(d)). Phosphorylation of ERK1/2 was increased in cells exposed to hypoxia, confirming hypoxic treatment of cells.^43^ Although pan ERK1/2 was found in sEVs, signals of phosphorylated ERK1/2 were below the detection limit. Expected sEV morphology, in particular the presence of a double lipid bilayer was confirmed with cryo-TEM (Figure 3(c)). Both Cryo-TEM and NTA have shown expected size range for the sEVs. These results indicated the suitability and correctness of the applied sEV preparation procedure.^41^ Altogether, based on protein and cryo-EM as qualitative, and NTA analysis as quantitative method these findings confirmed the successful isolation of N and H sEVs from DU145 cells.

### 3.3. Impact of hypoxic and normoxic DU145 sEVs on the SSGB model

**Figure 4.**
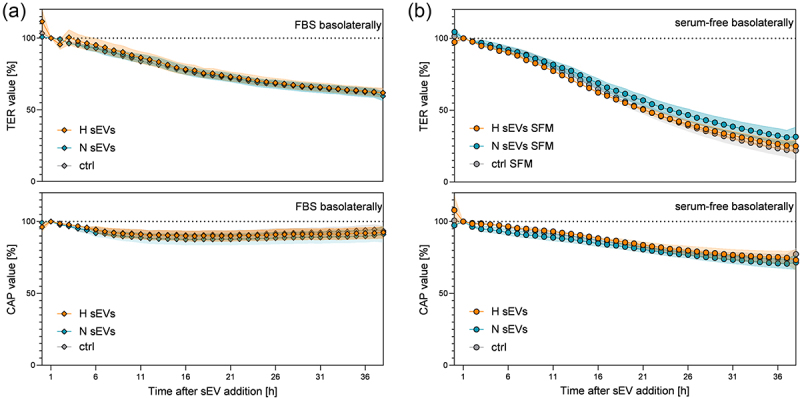
Barrier integrity after addition of DU145 sEvs on the basolateral side of the SSGB model (HTB-41 clone B2 cells on ThinCerts®). On the apical (salivary) side, media without serum were used, on the basolateral (blood) side either media with 10% FBS (a) or serum-free (b) media were applied. Transepithelial electrical resistance (TER) and capacitance of the cell layer (CAP) were recorded using a CellZscope device. Data normalized to data 1 h after media change (a,b) are shown as mean values ± SEM. *N* = 3–4, *n* = 8–12. Significant differences were observed between basolateral FBS vs. serum-free conditions, whereas sEV treatment exhibited no statically different effects. Significant differences are listed in table S5 (TER) and table S6 (CAP).

Impact of N and H sEVs on barrier integrity was measured continuously with the cellZscope measurement system. To remain physiologically more accurate and avoid contamination of FBS EVs in further analyses apical serum-free media were used in sEV experiments and basolaterally either serum-free or 10% FBS containing media were applied. In the more stabilized barrier model (10% FBS basolaterally), sEVs did not have significant effects on the physical barrier properties (H sEVs: 63,6 ± 3,2; N sEVs: 59,6 ± 3,2; ctrl: 61,9 ± 2,9% of initial TER value). Under serum-free, TER lowering conditions, N sEVs exhibited a slight improvement of barrier function (H sEVs: 24,9 ± 4,3; N sEVs: 31,4 ± 6,8; ctrl: 21,9 ± 6,5% of initial TER value), but overall, differences in TER and CAP observed upon basolateral addition of N and H sEV were not significantly different (Figure 4). However, significant differences were observed in all controls and treatments, when serum-free medium was applied basolaterally in comparison to FBS-containing medium (Table S5, Table S6). In conclusion, addition of sEVs from either normoxic or hypoxic conditions did not have detrimental or strengthening effects on TER or CAP of the SSGB model.

### 3.4. Molecular analysis data support serum dependency of the salivary gland barrier function, but also elucidate possible sEV regulated targets

**Figure 5.**
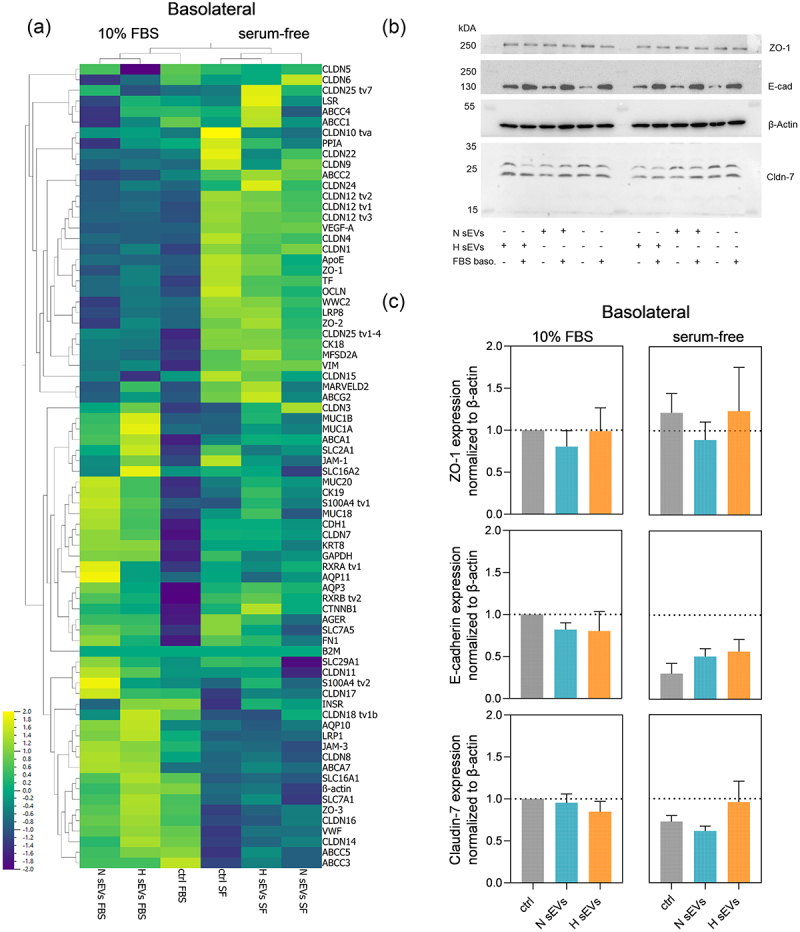
Changes at the molecular level after basolateral treatment of the SGGB model with H or N DU145 sEvs in serum-free or 10% FBS supplemented medium. (a) for the same samples mRNA expression of selected targets in HTB-41 clone B2 barrier after treatment with H or N DU145 sEvs for in either serum-free media or 10% FBS added basolaterally. *n* = 3, *N* = 3 pooled samples. Compressed legend. (b) Protein expression of E-cadherin, ZO-1, ß-actin and claudin-7 visualized by Western blotting. (c) Quantified protein expression levels of claudin-7, E-cadherin and zonula occludens-1 (ZO-1). Relative protein expression levels were determined by calculating relative density ratios to ß-actin and then normalized to 10% FBS control. Mean ± SEM, *n* = 3, *N* = 3 of pooled samples. E-cadherin expression between basolateral 10% FBS vs. serum-free control; *p* = 0.0439.

In order to reveal possible expression changes of barrier-relevant genes, SSGB samples after H or N sEV treatment and under different serum conditions were applied in an in-house developed high-throughput qPCR chip. Three main groups of co-regulated genes were identified (Figure 5(a), Table S7): A first group includes targets upregulated in serum-free conditions, including ZO-1 and the transporter proteins MRP1 and BCRP. Additionally, serum-dependent upregulation was observed for several claudins (CLDN1, CLDN4, CLDN9, CLDN12). In the second group targets down-regulated in serum-free conditions were found, such as JAM3, CLDN14 and CLDN18. The third main group were targets with increased expression after sEV treatment. In sEV exposed cells we observed upregulation of CLDN3, CK18, CK19 and aquaporins. Interestingly, this trend was seen mostly in samples with 10% FBS basolaterally, while in serum-free samples there was no difference between sEV treatment and controls for CK18 or AQP10. H and N sEVs treatment was, however, linked with the upregulation of several mucins (MUC1A, MUC1B, MUC20) in both serum conditions. Overall, the detected changes in gene expression support our previously observed changes in barrier integrity.

Furthermore, Western blots revealed a significant decrease of E-cadherin expression in serum-free media as compared to 10% FBS basolaterally (Figure 5(b,c)). This decrease was not detected at the mRNA level, indicating a post-translational regulatory mechanism. Besides controls, N sEV treatment showed a trend to downregulate ZO-1 expression in comparison to H sEV and untreated controls, both at protein and mRNA level.

In summary, changes observed in TER and CAP measurements before, were reflected by a number of barrier-relevant genes showing differential expression levels, both on mRNA and protein level.

### 3.5. Hypoxic sEVs derived from PCa cells increase the release of sEV sized particles in the salivary compartment

**Figure 6.**
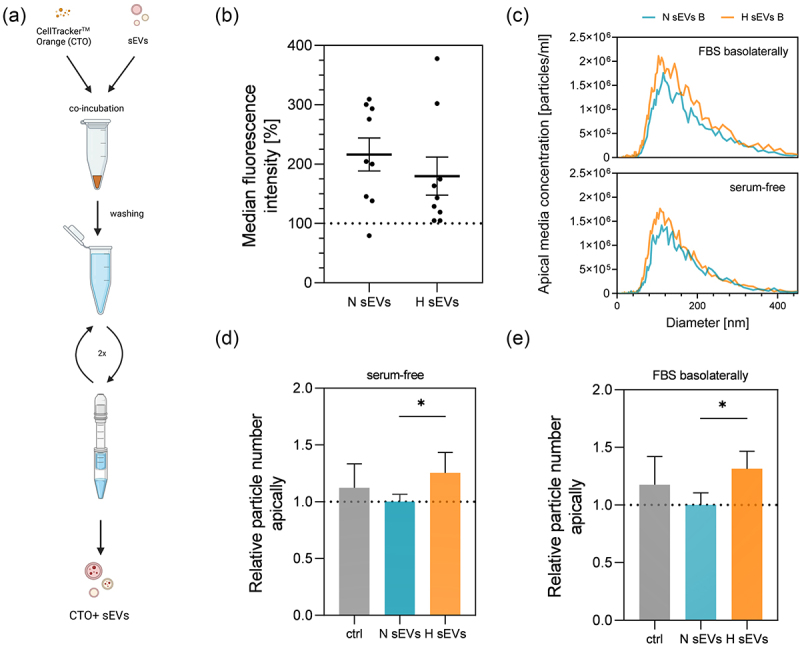
Uptake of CTO labeled hypoxia derived (H) and normoxia derived (N) sEvs measured with flow cytometer after 40 h incubation. (a) sEV labeling scheme with CellTracker^TM^ orange. (b) uptake of CTO+ H and N sEvs. Mean ± SEM *n* = 9, *N* = 3. (c) size distribution of sEV particles in the apical compartment after treatment with either H or N sEvs from the basolateral side in different serum conditions basolaterally. Data are represented as an average of three measurements, *n* = 3–4, *N* = 3–4. (d,e) sEV particles measured in the apical compartment with nanoparticle tracking analyzer 40 h after treatment with either H or N sEvs in the basolateral compartment in (d) serum-free or (e) 10% FBS and serum-free apically. Apical media collected after incubation period was subjected to a series of centrifugation steps as described in 2.5 to deplete any cells, apoptotic bodies, or larger vesicles. Data was normalized to normoxic sEV treatment. (d) Mean ± SD *n* = 11, *N* = 4 of pooled samples. (e) Mean ± SD, *n* = 8, *N* = 3 of pooled samples. Data was analyzed with one-way ANOVA followed by pairwise Holm-Šidak’s multiple comparison post hoc tests, (d) *p* = 0,0032, (e) *p* = 0.0056.

Next, the question whether DU145 sEVs could cross the SSGB model from the basolateral to the apical side was addressed. H and N sEVs were added into the basolateral media of the SSGB model and after 40 h incubation, apical media were collected. Media were depleted of cells and larger vesicles and particles by consecutive centrifugation steps (as described in Section 2.5) and analyzed by NTA. The size distribution of the measured particles from apical media corresponded to that of sEVs (Figure 6), while in the serum-free media as control no reliable number of particles could be detected (data not shown). Interestingly, addition of H sEVs led to an increase of apical sEV-like particles, both, in serum-free (+26% ± 17) and serum-containing basolateral media (+33% ± 12) as compared to treatment with N sEVs.

In order to discriminate if those additional particles represented H sEVs transported to the salivary compartment, or were released by the SSGB cells, we performed a series of experiments with CTO+ (fluorescently) labeled sEVs. Media labeling controls (as described in 2.4 and 2.8) were included to ensure that only sEV specific signals were recorded. First the uptake of N and H sEVs into SSGB cells was recorded with flow cytometry where median fluorescence intensity of cells in the CTO channel was normalized to media labeling control as negative control. Here, interestingly, no significant differences were observed in uptake between N and H sEV treatments. After 40 h, the apical media were processed as described before and apical samples were measured with the NTA’s fluorescence mode. In several experiments (*N* = 3) only sporadic fluorescent sEV-sized particles could be detected, leading to the conclusion that H sEVs do not permeate in significant numbers to the salivary compartment (data not shown). This result indicated, that H sEVs might have induced higher number of sEV sized particle secretion by the SSGB cells in comparison to N sEVs.

## 4. Discussion

The BSB is crucial in preventing noxious agents entry into the bloodstream, as well as transport of various biomolecules from blood to saliva, a process which is being taken advantage of in liquid biopsy applications e.g. sEV diagnostics from saliva.^4,44^ Although, there are some reports of sEVs crossing epithelial and endothelial biological barriers,^45–47^ not much is known so far about sEV interactions with the BSB. We aimed to test the potential effects and transport of sEVs from blood to the salivary compartment in an *in vitro* BSB model. As a source of sEVs the well characterized PCa cell line DU145 was chosen, as it originates from a brain metastasis and might therefore release sEVs able to compromise biological barriers.

Although some studies investigating the role of EVs in cell culture models of biological barriers have been conducted,^47–50^ to our knowledge this is the first report of sEV interaction with SSGB cells and in a BSB model setup.

Stable, cell line based BSB models are sparse and still in development,^1,36^ therefore, but also in general it is of great importance to test the suitability of the model for a given purpose. Serum exclusion in the apical compartment is important for physiological relevance of the utilized HTB-41 B2 *in vitro* model, as it represents the corresponding salivary compartment *in vivo*. Additionally, it enables more straightforward downstream analyses without introduced FBS sEVs, lipoproteins or larger protein complexes that are similar in size and density as sEVs released by cells.^51^ Therefore, although significant differences were observed in barrier tightness between serum-free and FBS containing media in the apical compartment (Figure 2), sEV experiments were carried out with serum-free conditions apically. With the more physiologically relevant, serum-free condition in the salivary compartment, we focused on testing different FBS compositions in the basolateral (blood) side. Optimization of experimental conditions for sEV studies revealed serum-dependent effects on the tightness of the SSGB model and the importance of serum sEVs (and most likely also other serum components) in the basolateral compartment of the model for maintaining barrier function (Figure 2). Serum-free, 10% FBS and 10% sEV depleted FBS conditions were compared. EV depletion from FBS has already been shown to affect the phenotype, proliferation, and EV release of treated cells,^52,53^ which corresponds with our observation of decreasing TER in this condition (Figure 2). For sEV depletion we utilized ultrafiltration, which has been shown to be an effective method to deplete FBS sEVs^52,54^. However, differences observed between FBS and sEV depleted FBS in barrier integrity (Figure 2) cannot exclude, that other small components of FBS are depleted in addition, which would make interpretations on DU145 sEV effects unfeasible, so we further focused on serum-free and 10% FBS conditions basolaterally.

The comparison of basolateral serum-free and FBS-containing conditions revealed a number of differently expressed genes, both at protein and mRNA level. Many genes important for maintaining paracellular barrier function, such as tight junction proteins, showed a regulation trend depending on the basolateral presence of serum (Figure 5).

The most abundant serum protein albumin can bind and transport other proteins, fatty acids, hormones, nutrients, and other molecules,^55^ and coating with cationic bovine serum albumin was used to deliver vesicles with siRNA *in vitro* and *in vivo*.^56^ Additionally, other FBS components like different globulins were proposed to have important role in EV delivery via the establishing of the so-called protein corona.^57^ Therefore, we tested both, serum-free and FBS containing media on the blood side for the sEV application, although it appeared that purified DU145 sEVs already exhibited a denser ring around the lipid bilayer of the sEV that could correspond to a protein corona (Figure 3c). Nevertheless, different FBS containing media enabled us to compare, but also to exclude possible media bias in comparison of N and H sEV effects on HTB-41 B2 barrier properties. In contrast to media composition (serum-free, FBS) no significant differences were observed in TER or CAP values after basolateral addition of H or N sEVs (Figure 4). However, in case of N sEV treatment a tendency for a less reduced TER was observed under serum-free conditions. This could correspond to reported effects of normoxic tumor EVs from oral squamous cell carcinoma cells, where those EVs stimulated γδ T-cell expansion and function, but the effect was diminished by hypoxic EV treatment.^57^ Another possible explanation for this effect could be attributed to EV signaling in general. EVs from different sources, among them also from PCa and in particular from DU145 cells, have been shown to carry important nutrients for cells and can have pro-survival properties.^58–61^ In this context, it could be speculated that in the complete absence of FBS the N sEVs acted as a nutrient source, while H sEVs due to different molecular compositions acted differently (Table S8). Although, it cannot be directly compared due to concentration and EV purification differences, a similar effect was observed in experiments presented in Figure 2, where the depletion of the EV-rich fraction in the FBS reduced barrier integrity when comparing with the media with complete FBS.

A number of tight junction associated or tight junction proteins such as ZO-1, ZO-2, Claudins (1, 4) and Occludin were differentially expressed depending on FBS content of the media. In case of Claudin-7, its downregulation was reported to be connected with more aggressive PCa,^62–65^ similarly, it was shown that E-cadherin loss can occur already in very early stages of benign hyperplasia.^66^ When Claudin-7 loss correlated with E-cadherin, this led to a more invasive phenotype in esophageal squamous carcinoma cells.^67^ In case of DU145 sEVs applied to the SSGB model, there was a slight decrease of Claudin-7 protein expression in the SSGB model after administration of H sEVs (0,67 ± 0,16) and to a lesser extent after N sEV (0,84 ± 0,08) treatment (Figure 5). In addition, a slight decrease of E-cadherin in sEV treated SSGB cells in FBS containing media was detected, whereas E-cadherin loss in serum-free conditions was attributed to serum depletion and was even lower under control conditions in comparison to sEV treated cells. In conclusion, the decrease of Claudin-7 and E-cadherin after sEV treatment under FBS containing conditions in the SSGB model seemed to reflect the known physiological biomarker situation more closer.

Another interesting target is S100A4 upregulated by sEV treatment in our model (1.1 to 3.4-fold). S100A4 is associated with the pathogenesis of different cancers, amongst them also PCa. In PCa, S100A4 was shown to promote cell proliferation and epithelial-to-mesenchymal transition (EMT) *in vitro*.^11^ In patients it was described as potential biomarker in plasma and biopsy samples^68,69^ and anti-S100A4 antibody therapy had a positive effect of PCa treatment in a mouse model.^69^ An additional cancer-associated target found at higher levels after sEV treatment was β-catenin. Higher expression of β-catenin was reported in EVs from hypoxic PCa cells^70^ and its upregulation in hypoxia was shown to have a role in PCa progression, where HIF-1α binding to β-catenin resulted in its nuclear translocation and androgen receptor activation.^71^

When uptaken by recipient cells, sEVs can alter cell function and behavior, including having a role in cancer development and pre-metastatic niche formation. It was shown that low oxygen concentration in the (tumor) microenvironment alters the expression profile of non-cancer and cancer cells, amongst them PCa sEVs,^10,58^ where H sEVs can increase cell invasion in comparison to N sEVs as examined via wound healing assays.^70^ H and N sEVs from the PCa cell line PC-3 (isolated from bone metastasis) exhibited different proteomes and in mouse models, H sEVs increased expression of EMT promoting proteins, selectively at pre-metastatic niche sites.^10^ In our experiments, uptake of H and N derived sEVs (Figure 6) occurred at a similar ratio and also TER was stable after sEV treatment (Figure 4). But interestingly, only H sEV treatment induced more particles in the apical salivary compartment of the SSGB model (Figure 6). Hypoxia is a known inducer of sEV secretion^31,32^ and H sEVs were shown to carry different metabolites in comparison to sEVs released under normoxic conditions.^72^ In our preliminary proteomic analysis of DU145 sEVs (Table S8), unique hypoxia targets were identified in H sEVs that were absent from N sEVs. The most abundant include hypoxia regulated biglycan,^72^ dihydropyrimidinase-related protein 5, which was connected with enhanced invasion and proliferation of PCa,^73^ and pentraxin-related protein PTX3, that was shown to have a role in acceleration of breast cancer metastasis when present in EVs.^74^ Therefore, it could be speculated that this hypoxia specific cargo in sEVs could contribute to the observed increased secretion of sEVs by the HTB-41 B2 cells.

To elucidate the origin of apically found EV-sized particles DU145 sEVs were labeled with CTO before application to the basolateral side. For sEV labeling we opted for an intraluminal dye, since due to the heterogeneous nature of sEVs and lack of cell specific markers on the sEVs reflecting their origin, it is particularly challenging to distinguish cancer and non-cancer-derived sEVs. But neither with fluorescence coupled NTA nor with the highly sensitive single-particle analysis method EVQuant,^69^ reliable numbers of CTO positive particles could be detected in the apical compartment (data not shown). Hence, it was hypothesized that the majority of the additionally detected particles in the apical compartment were indeed released by the SSGB cells. This could partially correspond to the proposed “EV-crine hypothesis”, where receiver cells release EVs with specific cargo as an answer to the donor cell EVs.^73^

The current study focused on DU145 PCa sEVs, utilizing hypoxia to mimic more disease relevant conditions (H sEVs) and to compare these to control N sEV treatment. Additional studies with sEVs from different tissue sources could be accomplished to investigate whether the hypoxia effects are PCa related or DU145 cell line specific. It is important to note that including different cell types for sEV experiments and draw conclusions from this comparison can be challenging. A major obstacle can be different basal media components used for the culture of the different cell lines to harvest the sEVs. These media components can be packaged into or be co-purified with vesicles.^74^ For example, one of the few benign prostate hyperplasia immortalized cell line PWR-1E is routinely cultivated in media with added growth factors that could have confounding effects when attached to the enriched sEVs.^75^ One option for future studies to test different effects of sEVs from PCa lines with different metastatic potential would be the use of LNCaP PCa cell line and it´s clones which possess different metastatic potential.^76^

In summary, N and H sEV were uptaken at similar ratio and had no significant effect on the barrier integrity. However, sEVs released under hypoxic conditions triggered the release of more sEV-sized particles in the SSGB model, an effect which was not described until now. Clearly, more experiments will be necessary to decipher the possible molecular mechanisms behind sEV induced vesicle release in target cells, and to understand their role in altering barrier signaling via sEV release.

## Supplementary Material

Supplementary Tables_12042024.docx
