# Supplementary material for: Effects of small extracellular vesicles derived from normoxia- and hypoxia-treated prostate cancer cells on the submandibular salivary gland epithelium in vitro
Source: Tissue Barriers. 2024 May 9;13(1):2347062. doi: 10.1080/21688370.2024.2347062 (PMC11875469; doi:10.1080/21688370.2024.2347062)
Supplement: Supplementary Tables_12042024.docx [file KTIB_A_2347062_SM4759.docx]

**Supplementary Tables**

Table S1

Characterisation of DU145 H and N EVs using NTA. Median size (nm) and zeta potential (mV) of EVs. Mean ± SD, N=3-4, n=3-4.

| *Small EVs* | *Median size (nm)* | *Zeta potential (mV)* |
| --- | --- | --- |
| DU145 N | 134.9±5.8 | -30.5±2.3 |
| DU145 H | 134.0±6 | -29.6±1.7 |

Table S2

List of antibodies for Western blotting. Alix, ALG-2-interacting protein X. CD81, Cluster of Differentiation 81. CD9, Cluster of Differentiation 9. GM130, cis-Golgi matrix protein, 130. ZO-1, Zonula occludens-1.

| *Target* | *Dilution* | *Isotype* | *Conjugate* | *Clone* | *Company* | *Catalogue number* |
| --- | --- | --- | --- | --- | --- | --- |
| Alix | 1:1000 | Mouse IgG1 | unconjugated | 3A9 | Cell Signaling Technology | 2171S |
| CD81 | 1:1000 | Mouse IgG1 | unconjugated | 1G2C6 | ProteinTech GmbH | 66866-1-Ig |
| CD9 | 1:1000 | Rabbit IgG | unconjugated | D3H4P | Cell Signaling Technology | 13403S |
| GM130 | 1:1000 | Rabbit IgG | unconjugated | Not applicable | ProteinTech GmbH | 11308-1-AP |
| ZO-1 | 1:100 | Rabbit IgG | unconjugated | ZMD.437 | Invitrogen | 40-2200 |
| E-cadherin | 1:1000 | Mouse | unconjugated | Not applicable | Santa cruz | SC-8426 |
| Claudin-7 | 1:125 | Rabbit | unconjugated | Not applicable | Invitrogen | 349100 |
| β-actin | 1:20000 | Mouse IgG1 | Peroxidase conjugate | AC-15 | Sigma-Aldrich | A3854 |
| Mouse IgG | 1:5000 | Horse | Peroxidase conjugate | Not applicable | Cell Signaling Technology | 7076 |
| Rabbit IgG | 1:5000 | Donkey | Peroxidase conjugate | F(ab′)2 | GE Healthcare UK Limited | LNA934V/AH |

Table S3

Statistical significance between TER values from figure 2. ns = p > 0.05, * = p < 0.05, ** p < 0.01, *** p < 0.001, *** p < 0.0001.

| Timepoint  \compared condition | A FBS, B FBS vs. A serum-free, B EV depleted FBS | A FBS, B FBS vs. A serum-free, B serum free | A FBS, B FBS vs. A serum-free, B FBS | A serum-free, B EV depleted FBS vs. A serum-free, B serum free | A serum-free, B EV depleted FBS vs. A serum-free, B FBS | A serum-free, B serum-free vs. A serum-free, B FBS | A serum free, B serum free vs. A and B EV depleted FBS |
| --- | --- | --- | --- | --- | --- | --- | --- |
| 1 to 13 | ns | ns | ns | ns | ns | ns | ns |
| 14 | ns | * | ns | ns | ns | ns | ns |
| 15 | ns | * | ns | ns | ns | ns | ns |
| 16 | ns | * | ns | ns | ns | ns | ns |
| 17 | * | ** | ns | ns | ns | ns | ns |
| 18 | * | ** | ns | ns | ns | * | ns |
| 19 | * | ** | ns | ns | ns | * | ns |
| 20 | * | ** | ns | ns | ns | ** | ns |
| 21 | * | ** | ns | ns | ns | ** | ns |
| 22 | * | ** | ns | ns | ns | ** | * |
| 23 | * | ** | ns | ns | ns | ** | ns |
| 24 | * | *** | ns | ns | * | ** | ** |
| 25 | * | *** | ns | ns | * | *** | ** |
| 26 | ** | *** | ns | ns | * | *** | ** |
| 27 | ** | *** | ns | ns | * | *** | ** |
| 28 | ** | *** | ns | ns | * | *** | ** |
| 29 | ** | *** | ns | ns | * | *** | ** |
| 30 | ** | *** | ns | * | ** | **** | ** |
| 31 | ** | *** | ns | * | * | **** | * |
| 32 | ** | *** | ns | * | * | **** | * |
| 33 | ** | **** | ns | * | * | **** | ns |
| 34 | ** | **** | ns | * | ** | **** | ns |
| 35 | ** | **** | ns | * | ** | **** | ns |
| 36 | ** | **** | ns | * | ** | **** | ns |
| 37 | ** | **** | ns | * | ** | **** | ns |
| 38 | ** | **** | ns | * | ** | **** | ns |
| 39 | *** | **** | * | ns | ** | *** | ns |

Table S4

Statistical significance between CAP values from figure 2. ns = p > 0.05, * = p < 0.05, ** p < 0.01.

| Timepoint  \compared condition | A FBS, B FBS vs. A serum-free, B EV depleted FBS | A FBS, B FBS vs. A serum-free, B serum-free | A FBS, B FBS vs. A serum-free, B FBS | A serum-free, B EV depleted FBS vs. A serum-free, B serum-free | A serum-free, B EV depleted FBS vs. A serum-free, B FBS | A serum-free, B serum-free vs. A serum-free, B FBS | A serum-free, B serum-free vs. A and B EV depleted FBS |
| --- | --- | --- | --- | --- | --- | --- | --- |
| 1 to 19 | ns | ns | ns | ns | ns | ns | ns |
| 20 to 28 | ns | ns | ns | * | ns | ns | ns |
| 29 | ns | ns | ns | ** | ns | ns | ns |
| 30 | ns | ns | ns | ** | ns | ns | ns |
| 31 | ns | ns | ns | * | ns | ns | ns |
| 32 | ns | ns | ns | ** | ns | ns | ns |
| 33 | ns | ns | ns | ** | ns | ns | ns |
| 34 | ns | ns | ns | ** | ns | * | ns |
| 35 | ns | ns | ns | * | ns | * | ns |
| 36 | ns | ns | ns | * | ns | * | ns |
| 37 | ns | ns | ns | ** | ns | * | ns |
| 38 | ns | ns | ns | * | ns | * | ns |
| 39 | ns | ns | ns | ns | ns | ns | ns |

Table S5

Statistical significance between TER values from figure 4. ns = p > 0.05, * = p < 0.05, ** p < 0.01, *** p < 0.001, *** p < 0.0001.

| Timepoint \ compared condition | H EVs serum-free vs. H sEVs FBS | H sEVs serum-free vs. N sEVs FBS | H sEVs serum-free vs. ctrl FBS | H sEVs FBS vs. N sEVs serum-free | H sEVs FBS vs. ctrl serum-free | N sEVs serum-free vs. N sEVs FBS | N sEVs serum-free vs. ctrl FBS | N sEVs FBS vs. ctrl serum-free | ctrl serum-free vs. Ctrl FBS |
| --- | --- | --- | --- | --- | --- | --- | --- | --- | --- |
| 1 to 12 | ns | ns | ns | ns | ns | ns | ns | ns | ns |
| 13 | ns | ns | * | ns | ns | ns | ns | ns | ns |
| 14 | ns | ns | ** | ns | ns | ns | ns | ns | ns |
| 15 | ns | * | *** | ns | ns | ns | ns | ns | ns |
| 16 | ns | * | *** | ns | ns | ns | ns | ns | ns |
| 17 | * | ** | **** | ns | ns | ns | ns | ns | * |
| 18 | * | ** | **** | ns | * | ns | ns | * | * |
| 19 | ** | ** | **** | ns | * | ns | ns | * | ** |
| 20 | ** | *** | **** | ns | ** | ns | ns | ** | ** |
| 21 | ** | *** | **** | ns | ** | ns | * | ** | ** |
| 22 | *** | *** | **** | * | ** | * | * | ** | *** |
| 23 | *** | **** | **** | * | *** | * | * | *** | *** |
| 24 | *** | **** | **** | * | *** | * | ** | *** | *** |
| 25 | **** | **** | **** | ** | *** | ** | ** | *** | *** |
| 26 | **** | **** | **** | ** | *** | ** | ** | *** | *** |
| 27 | **** | **** | **** | ** | *** | ** | ** | *** | *** |
| 28 | **** | **** | **** | ** | *** | ** | ** | *** | **** |
| 29 | **** | **** | **** | ** | **** | ** | ** | **** | **** |
| 30 | **** | **** | **** | ** | **** | ** | ** | **** | **** |
| 31 | **** | **** | **** | ** | **** | ** | ** | **** | **** |
| 32 | **** | **** | **** | ** | **** | ** | ** | **** | **** |
| 33 | **** | **** | **** | *** | **** | *** | *** | **** | **** |
| 34 | **** | **** | **** | *** | **** | *** | *** | **** | **** |
| 35 | **** | **** | **** | *** | **** | *** | *** | **** | **** |
| 36 | **** | **** | **** | *** | **** | *** | *** | **** | **** |
| 37 | **** | **** | **** | *** | **** | *** | *** | **** | **** |
| 38 | **** | **** | **** | *** | **** | *** | *** | **** | **** |
| 39 | **** | **** | **** | * | ** | * | * | ** | ** |

| Timepoint \ compared condition | H sEVs serum-free vs. N sEVs serum-free | H sEVs serum-free vs. ctrl serum-free | N sEVs serum-free vs. ctrl serum-free |  | Timepoint \ compared condition | H sEVs FBS vs. N sEVs FBS | H sEVs FBS vs. ctrl FBS | N sEVs FBS vs. ctrl FBS |
| --- | --- | --- | --- | --- | --- | --- | --- | --- |
| 1 to 39 | ns | ns | ns |  | 1 to 39 | ns | ns | ns |

Table S6

Statistical significance between CAP values from figure 4. ns = p > 0.05, * = p < 0.05, ** p < 0.01, *** p < 0.001, *** p < 0.0001.

| Timepoint \ compared condition | H EVs serum-free vs. H sEVs FBS | H sEVs serum-free vs. N sEVs FBS | H sEVs serum-free vs. ctrl FBS | H sEVs FBS vs. N sEVs serum-free | H sEVs FBS vs. ctrl serum-free | N sEVs serum-free vs. N sEVs FBS | N sEVs serum-free vs. ctrl FBS | N sEVs FBS vs. ctrl serum-free | ctrl serum-free vs. Ctrl FBS |
| --- | --- | --- | --- | --- | --- | --- | --- | --- | --- |
| 1 to 25 | ns | ns | ns | ns | ns | ns | ns | ns | ns |
| 26 | ns | ns | ns | ns | ns | ns | * | ns | ns |
| 27 | ns | ns | ns | ns | ns | ns | * | ns | ns |
| 28 | ns | ns | ns | ns | ns | ns | * | ns | * |
| 29 | ns | ns | ns | ns | ns | ns | * | ns | * |
| 30 | ns | ns | ns | * | ns | ns | ** | ns | * |
| 31 | ns | ns | ns | * | ns | ns | ** | ns | * |
| 32 | ns | ns | ns | * | ns | ns | ** | ns | * |
| 33 | ns | ns | * | * | ns | * | ** | ns | * |
| 34 | ns | ns | * | * | ns | * | ** | ns | * |
| 35 | ns | ns | * | * | * | * | ** | ns | ** |
| 36 | ns | ns | * | * | * | * | ** | ns | ** |
| 37 | ns | ns | * | * | * | * | ** | ns | ** |
| 38 | ns | ns | * | * | * | * | ** | ns | ** |
| 39 | ns | ns | ns | ns | ns | ns | * | ns | ns |

| Timepoint \ compared condition | H sEVs serum-free vs. N sEVs serum-free | H sEVs serum-free vs. ctrl serum-free | N sEVs serum-free vs. ctrl serum-free |  | Timepoint \ compared condition | H sEVs FBS vs. N sEVs FBS | H sEVs FBS vs. ctrl FBS | N sEVs FBS vs. ctrl FBS |
| --- | --- | --- | --- | --- | --- | --- | --- | --- |
| 1 to 39 | ns | ns | ns |  | 1 to 39 | ns | ns | ns |

Table S7

List of BBB chip average values shown as mean ± SD. N=3, n=3. Statistical significance from figure 6. * = p < 0.05 vs. H sEVs 10% FBS, # = p < 0.05 vs. N sEVs 10% FBS, § = p < 0.05 vs. ctrl serum-free, $ = p < 0.05 vs. ctrl 10% FBS.

| Fold-change | H sEVs, serum-free B | | | H sEVs, 10% FBS B | | | N sEVs, serum-free B | | | N sEVs, 10% FBS B | | | ctrl, serum-free B | | | ctrl, 10% FBS B | | |
| --- | --- | --- | --- | --- | --- | --- | --- | --- | --- | --- | --- | --- | --- | --- | --- | --- | --- | --- |
| PPIA | 1.05 | ± | 0.68 | 0.96 | ± | 0.61 | 0.92 | ± | 0.42 | 0.79 | ± | 0.36 | 1.24 | ± | 0.77 | 1.00 | ± | 0.00 |
| ß-actin | 0.62 | ± | 0.47 | 1.04 | ± | 0.58 | 0.47 | ± | 0.17 | 0.86 | ± | 0.31 | 0.59 | ± | 0.29 | 1.00 | ± | 0.00 |
| GAPDH | 1.28 | ± | 0.98 | 1.62 | ± | 0.76 | 1.32 | ± | 0.69 | 1.63 | ± | 1.24 | 1.50 | ± | 0.90 | 1.00 | ± | 0.00 |
| B2M | 1.00 | ± | 0.00 | 1.00 | ± | 0.00 | 1.00 | ± | 0.00 | 1.00 | ± | 0.00 | 1.00 | ± | 0.00 | 1.00 | ± | 0.00 |
| CLDN1 | 1.57 | ± | 0.29 | 1.23 | ± | 0.17 | 1.68 | ± | 0.45 | 1.08 | ± | 0.37 | 1.71 | ± | 0.52 | 1.00 | ± | 0.00 |
| CLDN3 | 2.80 | ± | 3.32 | 3.58 | ± | 5.28 | 4.16 | ± | 6.52 | 2.51 | ± | 3.28 | 1.39 | ± | 1.46 | 1.00 | ± | 0.00 |
| CLDN4 | 3.08 | ± | 1.16 | 1.13 | ± | 0.17 | 2.81 | ± | 1.29 | 1.26 | ± | 0.72 | 4.00 | ± | 1.41 | 1.00 | ± | 0.00 |
| CLDN5 | 0.87 | ± | 0.61 | 0.48 | ± | 0.59 | 0.93 | ± | 0.42 | 0.97 | ± | 0.50 | 0.93 | ± | 1.19 | 1.00 | ± | 0.00 |
| CLDN6 | 0.84 | ± | 0.03 | 0.62 | ± | 0.32 | 1.24 | ± | 0.74 | 0.46 | ± | 0.30 | 0.77 | ± | 0.39 | 1.00 | ± | 0.00 |
| CLDN7 | 1.48 | ± | 1.16 | 1.62 | ± | 1.03 | 1.40 | ± | 0.98 | 1.74 | ± | 1.47 | 1.48 | ± | 1.15 | 1.00 | ± | 0.00 |
| CLDN8 | 0.80 | ± | 0.30 | 1.43 | ± | 0.30 | 0.67 | ± | 0.20 | 1.51 | ± | 1.04 | 0.74 | ± | 0.41 | 1.00 | ± | 0.00 |
| CLDN9 | 1.31 | ± | 0.83 | 1.09 | ± | 1.36 | 2.00 | ± | 1.57 | 0.93 | ± | 0.58 | 2.37 | ± | 1.81 | 1.00 | ± | 0.00 |
| CLDN10 tva | 2.05 | ± | 2.49 | 3.26 | ± | 2.44 | 0.33 | ± | 0.56 | 2.55 | ± | 4.08 | 14.27 | ± | 20.52 | 1.00 | ± | 0.00 |
| CLDN11 | 1.03 | ± | 0.74 | 1.20 | ± | 0.35 | 0.73 | ± | 0.23 | 1.35 | ± | 0.93 | 1.00 | ± | 0.80 | 1.00 | ± | 0.00 |
| CLDN12 tv1 | 1.97 | ± | 0.82 | 1.19 | ± | 0.27 | 1.78 | ± | 0.60 | 1.11 | ± | 0.38 | 2.07 | ± | 0.74 | 1.00 | ± | 0.00 |
| CLDN12 tv2 | 2.34 | ± | 0.53 | 1.12 | ± | 0.24 | 2.07 | ± | 0.45 | 1.03 | ± | 0.34 | 2.58 | ± | 1.05 | 1.00 | ± | 0.00 |
| CLDN12 tv3 | 2.67 | ± | 1.07 | 1.18 | ± | 0.26 | 2.53 | ± | 0.88 | 1.19 | ± | 0.54 | 3.03 | ± | 1.41 | 1.00 | ± | 0.00 |
| CLDN14 | 0.44 | ± | 0.43 | 1.15 | ± | 0.65 | 0.54 | ± | 0.94 | 0.81 | ± | 0.92 | 0.28 | ± | 0.25 | 1.00 | ± | 0.00 |
| CLDN15 | 1.25 | ± | 0.36 | 0.70 | ± | 0.06 | 1.03 | ± | 0.16 | 0.93 | ± | 0.19 | 1.42 | ± | 0.44 | 1.00 | ± | 0.00 |
| CLDN16 | 0.76 | ± | 0.11 | 1.12 | ± | 0.36 | 0.83 | ± | 0.29 | 1.04 | ± | 0.28 | 0.73 | ± | 0.11 | 1.00 | ± | 0.00 |
| CLDN17 | 0.80 | ± | 0.18 | 1.02 | ± | 0.40 | 0.76 | ± | 0.31 | 1.32 | ± | 1.06 | 0.58 | ± | 0.25 | 1.00 | ± | 0.00 |
| CLDN18 tv1b | 0.18 | ± | 0.19 | 1.36 | ± | 0.27 | 0.69 | ± | 0.06 | 0.51 | ± | 0.38 | 0.20 | ± | 0.18 | 1.00 | ± | 0.00 |
| CLDN22 | 1.03 | ± | 0.24 | 0.84 | ± | 0.19 | 1.53 | ± | 1.57 | 0.89 | ± | 1.02 | 2.23 | ± | 1.76 | 1.00 | ± | 0.00 |
| CLDN24 | 2.43 | ± | 2.57 | 1.16 | ± | 0.89 | 1.68 | ± | 1.35 | 0.94 | ± | 0.75 | 1.55 | ± | 0.68 | 1.00 | ± | 0.00 |
| JAM-1 | 1.12 | ± | 0.16 | 1.27 | ± | 0.39 | 1.05 | ± | 0.10 | 1.10 | ± | 0.25 | 1.34 | ± | 0.37 | 1.00 | ± | 0.00 |
| JAM-3 | 0.51 | ± | 0.09^*,#^ | 1.39 | ± | 0.31 | 0.36 | ± | 0.22^*,#^ | 1.50 | ± | 0.39 | 0.56 | ± | 0.22^*,#^ | 1.00 | ± | 0.00 |
| ZO-1 | 1.58 | ± | 0.37 | 1.15 | ± | 0.41 | 1.33 | ± | 0.27 | 0.97 | ± | 0.20 | 1.73 | ± | 0.58 | 1.00 | ± | 0.00 |
| ZO-2 | 1.40 | ± | 0.32 | 1.07 | ± | 0.35 | 1.21 | ± | 0.40 | 0.91 | ± | 0.12 | 1.32 | ± | 0.37 | 1.00 | ± | 0.00 |
| ZO-3 | 0.79 | ± | 0.04 | 1.10 | ± | 0.16 | 0.84 | ± | 0.26 | 1.01 | ± | 0.42 | 0.74 | ± | 0.25 | 1.00 | ± | 0.00 |
| VWF | 0.49 | ± | 0.33 | 1.13 | ± | 0.70 | 0.47 | ± | 0.27 | 0.96 | ± | 0.36 | 0.25 | ± | 0.07 | 1.00 | ± | 0.00 |
| SLC2A1 | 1.11 | ± | 0.05 | 1.44 | ± | 0.12 | 1.16 | ± | 0.22 | 1.20 | ± | 0.21 | 1.30 | ± | 0.19 | 1.00 | ± | 0.00 |
| OCLN | 1.84 | ± | 0.13 | 1.24 | ± | 0.24^§^ | 1.63 | ± | 0.19 | 1.16 | ± | 0.32^§^ | 2.28 | ± | 0.74 | 1.00 | ± | 0.00^§^ |
| ABCC1 | 1.08 | ± | 0.73 | 0.85 | ± | 0.53 | 0.87 | ± | 0.51 | 0.73 | ± | 0.44 | 0.89 | ± | 0.42 | 1.00 | ± | 0.00 |
| ABCC2 | 2.05 | ± | 0.57^#^ | 0.65 | ± | 0.27 | 1.78 | ± | 0.65 | 0.56 | ± | 0.07 | 1.64 | ± | 0.91 | 1.00 | ± | 0.00 |
| ABCC3 | 0.66 | ± | 0.07^$^ | 0.83 | ± | 0.19 | 0.60 | ± | 0.14^$^ | 0.84 | ± | 0.22 | 0.55 | ± | 0.11^$^ | 1.00 | ± | 0.00 |
| ABCC4 | 1.12 | ± | 0.39 | 0.99 | ± | 0.58 | 0.86 | ± | 0.47 | 0.81 | ± | 0.26 | 0.97 | ± | 0.24 | 1.00 | ± | 0.00 |
| ABCC5 | 0.69 | ± | 0.54 | 0.97 | ± | 0.59 | 0.57 | ± | 0.39 | 0.87 | ± | 0.44 | 0.46 | ± | 0.19 | 1.00 | ± | 0.00 |
| ABCG2 | 2.81 | ± | 2.60 | 1.54 | ± | 0.88 | 1.39 | ± | 0.59 | 1.06 | ± | 0.51 | 2.32 | ± | 0.54 | 1.00 | ± | 0.00 |
| MARVELD2 | 1.28 | ± | 0.08 | 1.17 | ± | 0.49 | 1.06 | ± | 0.13 | 1.01 | ± | 0.15 | 1.21 | ± | 0.38 | 1.00 | ± | 0.00 |
| SLC7A1 | 0.85 | ± | 0.30 | 1.14 | ± | 0.59 | 0.68 | ± | 0.20 | 1.00 | ± | 0.22 | 0.77 | ± | 0.12 | 1.00 | ± | 0.00 |
| SLC29A1 | 1.08 | ± | 0.74 | 1.06 | ± | 0.17 | 0.81 | ± | 0.51 | 1.16 | ± | 0.78 | 1.09 | ± | 0.73 | 1.00 | ± | 0.00 |
| INSR | 0.94 | ± | 0.23 | 0.99 | ± | 0.18 | 0.93 | ± | 0.36 | 0.89 | ± | 0.18 | 0.85 | ± | 0.06 | 1.00 | ± | 0.00 |
| SLC7A5 | 1.28 | ± | 0.79 | 1.39 | ± | 0.06 | 1.08 | ± | 0.63 | 1.47 | ± | 1.00 | 1.51 | ± | 1.05 | 1.00 | ± | 0.00 |
| LRP1 | 0.75 | ± | 0.04 | 1.38 | ± | 0.14 | 0.76 | ± | 0.24 | 1.26 | ± | 0.42 | 0.74 | ± | 0.25 | 1.00 | ± | 0.00 |
| LRP8 | 1.98 | ± | 0.62 | 1.07 | ± | 0.11 | 1.53 | ± | 0.27 | 0.85 | ± | 0.24 | 1.96 | ± | 0.45 | 1.00 | ± | 0.00 |
| SLC16A1 | 0.69 | ± | 0.19 | 1.14 | ± | 0.47 | 0.65 | ± | 0.22 | 0.98 | ± | 0.19 | 0.65 | ± | 0.11 | 1.00 | ± | 0.00 |
| SLC16A2 | 1.03 | ± | 0.42 | 1.22 | ± | 0.20 | 0.90 | ± | 0.28 | 0.99 | ± | 0.29 | 1.05 | ± | 0.50 | 1.00 | ± | 0.00 |
| TF | 2.06 | ± | 0.71 | 1.45 | ± | 0.35 | 2.02 | ± | 0.15 | 1.27 | ± | 0.49 | 2.53 | ± | 0.74 | 1.00 | ± | 0.00 |
| VEGF-A | 3.85 | ± | 1.75 | 1.05 | ± | 0.16 | 3.94 | ± | 1.74 | 0.91 | ± | 0.21 | 4.40 | ± | 1.45 | 1.00 | ± | 0.00 |
| LSR | 1.25 | ± | 0.51 | 1.04 | ± | 0.41 | 0.97 | ± | 0.34 | 0.86 | ± | 0.22 | 0.96 | ± | 0.25 | 1.00 | ± | 0.00 |
| WWC2 | 1.41 | ± | 0.10 | 1.04 | ± | 0.29 | 1.26 | ± | 0.19 | 0.87 | ± | 0.12 | 1.45 | ± | 0.37 | 1.00 | ± | 0.00 |
| KRT8 | 2.01 | ± | 1.42 | 2.75 | ± | 2.38 | 1.68 | ± | 1.80 | 2.79 | ± | 3.51 | 1.98 | ± | 2.30 | 1.00 | ± | 0.00 |
| CK18 | 7.03 | ± | 9.38 | 2.98 | ± | 2.77 | 5.99 | ± | 7.02 | 3.10 | ± | 3.70 | 6.97 | ± | 7.43 | 1.00 | ± | 0.00 |
| CK19 | 2.66 | ± | 1.63 | 2.74 | ± | 2.20 | 1.89 | ± | 1.96 | 3.60 | ± | 4.52 | 1.62 | ± | 1.56 | 1.00 | ± | 0.00 |
| AQP3 | 3.01 | ± | 3.25 | 2.52 | ± | 1.55 | 2.71 | ± | 2.35 | 3.32 | ± | 4.16 | 2.69 | ± | 2.95 | 1.00 | ± | 0.00 |
| AQP10 | 0.24 | ± | 0.30 | 3.64 | ± | 1.57 | 0.75 | ± | 0.94 | 3.04 | ± | 3.20 | 0.66 | ± | 0.75 | 1.00 | ± | 0.00 |
| AQP11 | 1.00 | ± | 1.16 | 2.27 | ± | 0.14 | 2.09 | ± | 2.55 | 6.33 | ± | 6.58 | 2.48 | ± | 2.93 | 1.00 | ± | 0.00 |
| MUC1A | 1.45 | ± | 1.10 | 3.24 | ± | 3.33 | 1.19 | ± | 1.11 | 2.05 | ± | 1.59 | 0.85 | ± | 0.54 | 1.00 | ± | 0.00 |
| MUC1B | 2.29 | ± | 1.47 | 3.93 | ± | 4.35 | 1.33 | ± | 1.10 | 2.49 | ± | 2.44 | 1.08 | ± | 0.78 | 1.00 | ± | 0.00 |
| MUC18 | 2.33 | ± | 2.28 | 2.37 | ± | 1.10 | 1.23 | ± | 0.99 | 2.98 | ± | 3.93 | 1.77 | ± | 1.92 | 1.00 | ± | 0.00 |
| MUC20 | 2.53 | ± | 2.14 | 2.80 | ± | 2.54 | 2.12 | ± | 2.54 | 3.72 | ± | 4.79 | 1.84 | ± | 2.17 | 1.00 | ± | 0.00 |
| CDH1 | 2.28 | ± | 2.53 | 2.63 | ± | 1.95 | 2.05 | ± | 2.35 | 3.21 | ± | 3.70 | 2.28 | ± | 2.71 | 1.00 | ± | 0.00 |
| CTNNB1 | 4.41 | ± | 3.67 | 2.84 | ± | 2.37 | 2.93 | ± | 3.17 | 3.10 | ± | 3.45 | 3.08 | ± | 3.04 | 1.00 | ± | 0.00 |
| VIM | 3.94 | ± | 4.14 | 2.32 | ± | 2.18 | 3.87 | ± | 5.16 | 2.02 | ± | 2.21 | 3.69 | ± | 4.43 | 1.00 | ± | 0.00 |
| FN1 | 2.63 | ± | 2.93 | 2.48 | ± | 2.08 | 1.91 | ± | 1.90 | 3.25 | ± | 4.20 | 2.90 | ± | 3.56 | 1.00 | ± | 0.00 |
| S100A4 tv1 | 1.88 | ± | 1.30 | 2.17 | ± | 0.82 | 1.29 | ± | 0.93 | 2.81 | ± | 3.43 | 0.96 | ± | 0.61 | 1.00 | ± | 0.00 |
| S100A4 tv2 | 1.48 | ± | 1.84 | 1.19 | ± | 0.71 | 0.66 | ± | 0.67 | 2.76 | ± | 4.23 | 0.67 | ± | 0.79 | 1.00 | ± | 0.00 |
| CLDN25 tv1-4 | 5.75 | ± | 6.27 | 2.87 | ± | 3.17 | 4.71 | ± | 6.17 | 3.13 | ± | 3.92 | 5.88 | ± | 8.09 | 1.00 | ± | 0.00 |
| CLDN25 tv7 | 4.32 | ± | 5.02 | 0.48 | ± | 0.42 | 2.22 | ± | 3.41 | 2.29 | ± | 2.42 | 1.15 | ± | 0.81 | 1.00 | ± | 0.00 |
| ABCA1 | 1.93 | ± | 1.45 | 2.69 | ± | 2.86 | 1.90 | ± | 2.36 | 2.21 | ± | 2.20 | 1.65 | ± | 2.18 | 1.00 | ± | 0.00 |
| ABCA7 | 1.27 | ± | 1.04 | 2.87 | ± | 3.20 | 0.94 | ± | 1.02 | 2.96 | ± | 3.50 | 0.90 | ± | 1.18 | 1.00 | ± | 0.00 |
| ApoE | 30.93 | ± | 37.08 | 2.99 | ± | 3.67 | 16.23 | ± | 16.84 | 4.45 | ± | 6.26 | 38.63 | ± | 52.99 | 1.00 | ± | 0.00 |
| RXRA tv1 | 2.06 | ± | 1.68 | 2.31 | ± | 1.84 | 2.57 | ± | 3.66 | 4.31 | ± | 5.94 | 2.36 | ± | 3.11 | 1.00 | ± | 0.00 |
| RXRB tv2 | 3.62 | ± | 4.10 | 2.99 | ± | 3.29 | 2.91 | ± | 3.68 | 3.44 | ± | 4.42 | 3.31 | ± | 4.62 | 1.00 | ± | 0.00 |
| MFSD2A | 5.55 | ± | 5.41 | 2.03 | ± | 1.79 | 4.27 | ± | 4.50 | 2.30 | ± | 2.65 | 4.45 | ± | 4.91 | 1.00 | ± | 0.00 |
| AGER | 3.52 | ± | 2.59 | 3.47 | ± | 4.32 | 2.14 | ± | 2.29 | 3.50 | ± | 4.32 | 4.25 | ± | 6.30 | 1.00 | ± | 0.00 |

Table S8

List of proteins found in either H or N EVs from most abundant to least abundant ones. In total 3093 protein were identified, from that 53 were unique for H and 19 were unique for N EVs.

|  | **Proteins found only in H DU145 EVs** | **Proteins found only in N DU145 EVs** |
| --- | --- | --- |
| 1 | Biglycan | A disintegrin and metalloproteinase with thrombospondin motifs 1 |
| 2 | Dihydropyrimidinase-related protein 5 | DNA-directed RNA polymerase I subunit RPA1 |
| 3 | Pentraxin-related protein PTX3 | Cysteine-rich secretory protein LCCL domain-containing 2 |
| 4 | Aconitate hydratase, mitochondrial | tRNA (guanine(26)-N(2))-dimethyltransferase |
| 5 | Insulin-like growth factor-binding protein 3 | DNA-directed RNA polymerase III subunit RPC3 |
| 6 | RalBP1-associated Eps domain-containing protein 2 | Mammalian ependymin-related protein 1 |
| 7 | Cyclin-Y | Calcium-binding protein 39-like |
| 8 | Mucin-5B | Serine protease hepsin |
| 9 | Receptor expression-enhancing protein 6 | Vacuolar protein sorting-associated protein 37D |
| 10 | U5 small nuclear ribonucleoprotein 40 kDa protein | Phosphoglucomutase-like protein 5 |
| 11 | Nucleotide exchange factor SIL1 | Heme-binding protein 2 |
| 12 | Apolipoprotein D | HAUS augmin-like complex subunit 4 |
| 13 | D-aminoacyl-tRNA deacylase 1 | Elongation factor G, mitochondrial |
| 14 | 1-phosphatidylinositol 4,5-bisphosphate phosphodiesterase gamma-1 | 5-oxoprolinase |
| 15 | U4/U6 small nuclear ribonucleoprotein Prp31 | Tissue-type plasminogen activator |
| 16 | DNA-3-methyladenine glycosylase | Collagenase 3 |
| 17 | Isocitrate dehydrogenase [NADP], mitochondrial | Follistatin |
| 18 | Mucin-5AC | Neural cell adhesion molecule 2 |
| 19 | Heparan-sulfate 6-O-sulfotransferase 1 | Pleckstrin homology domain-containing family A member 7 |
| 20 | Protocadherin-1 |  |
| 21 | tRNA (guanine(37)-N1)-methyltransferase |  |
| 22 | Inorganic pyrophosphatase 2, mitochondrial |  |
| 23 | COP9 signalosome complex subunit 7a |  |
| 24 | 3-hydroxyacyl-CoA dehydrogenase type-2 |  |
| 25 | Deoxycytidine kinase |  |
| 26 | Succinyl-CoA:3-ketoacid coenzyme A transferase 1, mitochondrial |  |
| 27 | Laminin subunit alpha-4 |  |
| 28 | Isocitrate dehydrogenase [NAD] subunit alpha, mitochondrial |  |
| 29 | N-acylneuraminate cytidylyltransferase |  |
| 30 | Medium-chain specific acyl-CoA dehydrogenase, mitochondrial |  |
| 31 | Hydroxyacyl-coenzyme A dehydrogenase, mitochondrial |  |
| 32 | Hexokinase-2 |  |
| 33 | Nuclear pore complex protein Nup160 |  |
| 34 | Transcription activator BRG1 |  |
| 35 | Golgin subfamily A member 4 |  |
| 36 | Glypican-4 |  |
| 37 | COMM domain-containing protein 9 |  |
| 38 | Phosphorylase b kinase regulatory subunit beta |  |
| 39 | TBC1 domain family member 17 |  |
| 40 | E3 ubiquitin-protein ligase MYCBP2 |  |
| 41 | Growth/differentiation factor 11 |  |
| 42 | Leucine-rich repeat LGI family member 2 |  |
| 43 | Lon protease homolog, mitochondrial |  |
| 44 | Plastin-2 |  |
| 45 | DNA-directed RNA polymerase III subunit RPC2 |  |
| 46 | Delta-1-pyrroline-5-carboxylate synthase |  |
| 47 | Bone marrow stromal antigen 2 |  |
| 48 | Myotubularin-related protein 1 |  |
| 49 | 5-azacytidine-induced protein 2 |  |
| 50 | ADAMTS-like protein 3 |  |
| 51 | Ubiquitin-like modifier-activating enzyme ATG7 |  |
| 52 | Phosphatidylinositol 3-kinase regulatory subunit beta |  |
| 53 | Cordon-bleu protein-like 1 |  |
